# Evolutionary dynamics of autosomal-heterosomal rearrangements in a multiple-X chromosome system of tiger beetles (Cicindelidae)

**DOI:** 10.1186/1471-2148-7-158

**Published:** 2007-09-06

**Authors:** José Galián, Sónia JR Proença, Alfried P Vogler

**Affiliations:** 1 Área de Biología Animal, Departamento de Zoología y Antropología Física, Universidad de Murcia, Apdo 4021, Murcia 30071, Spain; 2 Centro de Biologia Ambiental/Departamento de Zoologia e Antropologia, Faculdade de Ciências, Universidade de Lisboa, Campo Grande, Bloco C2 – 3° Piso, 1700 Lisboa, Portugal; 3 Department of Entomology, Natural History Museum, London, SW7 5BD, UK; 4 Department of Biology, Imperial College London, Silwood Park Campus, Ascot, Berkshire, SL5 7PY, UK

## Abstract

**Background:**

Genetic systems involving multiple X chromosomes have arisen repeatedly in sexually reproducing animals. Tiger beetles (Cicindelidae) exhibit a phylogenetically ancient multiple-X system typically consisting of 2–4 X chromosomes and a single Y. Because recombination rates are suppressed in sex chromosomes, changes in their numbers and movement of genes between sex chromosomes and autosomes, could have important consequences for gene evolution and rates of speciation induced by these rearrangements. However, it remains unclear how frequent these rearrangements are and which genes are affected.

**Results:**

Karyotype analyses were performed for a total of 26 North American species in the highly diverse genus *Cicindela*, tallying the number of X chromosomes and autosomes during mitosis and meiosis. The chromosomal location of the ribosomal rRNA gene cluster (rDNA) was used as an easily scored marker for genic turnover between sex chromosomes or autosomes. The findings were assessed in the light of a recent phylogenetic analysis of the group. While autosome numbers remained constant throughout the lineage, sex chromosome numbers varied. The predominant karyotype was n = 9+X_1_X_2_X_3_Y which was also inferred to be the ancestral state, with several changes to X_1_X_2_Y and X_1_X_2_X_3_X_4_Y confined to phylogenetically isolated species. The total (haploid) numbers of rDNA clusters varied between two, three, and six (in one exceptional case), and clusters were localized either on the autosomes, the sex chromosomes, or both. Transitions in rDNA localization and in numbers of rDNA clusters varied independently of each other, and also independently of changes in sex chromosome numbers.

**Conclusion:**

Changes of X chromosome numbers and transposition of the rDNA locus (and presumably other genes) between autosomes and sex chromosomes in *Cicindela *occur frequently, and are likely to be the result of fusions or fissions between X chromosomes, rather than between sex chromosomes and autosomes. Yet, translocations between sex chromosomes and autosomes appear to be common, as indicated by the patterns of rDNA localization. Rearranged karyotypes involving multiple sex chromosomes would reduce recombination, and hybrid dysgenesis selects against polymorphic populations. Hence, the high frequency of these rearrangements could be a cause of the great species diversity in *Cicindela*.

## Background

Chromosomal rearrangements have been described in many groups of animals, distinguishing even very closely related species and populations [1-4]. As rearrangements would change the position of genes, generating new linkage groups that may lead to an increase of genetic differentiation between populations, they are expected to restrict reproductive compatibility and eventually promote speciation. Chromosomal rearrangements produce unbalanced gametes which have been thought to be the primary effect of rearrangements in speciation because of the diminished fecundity or viability of the heterozygotes [5]. However, this notion of 'hybrid-dysfunction' as a major cause of karyotype-mediated speciation may not be an effective isolation mechanism [1,6,7]. More recently, it is being realized that the primary effect of chromosomal rearrangements is the reduced recombination between genomes, favoring 'suppressed-recombination' scenarios for the evolution of reproductive isolation following chromosomal rearrangements [1,6,8].

Recombination rates vary widely depending on the genomic position and the linkage with genes under selection, affecting the tempo and mode of gene evolution [e.g. [7,9,10]]. Specifically, recombination is greatly reduced in sex-chromosomal linkage groups, because of the lack of a homologous chromosome in the heterogametic sex, or it is prevented altogether where sex chromosomes are achiasmatic in one of the sexes. In addition, due to the hemizygosity of the X chromosome (or the W chromosome in ZW systems with heterogametic females), recessive mutations associated with the sex chromosomes are exposed to selection in the heterogametic sex [11]. Hence, as [12] pointed out, the origination of new X chromosomes leads to fixation of a great deal of heterozygosity (while new Y chromosomes lead to the rapid degradation of gene content; see [13]). As the result of reduced recombination, genetic barriers to gene flow may arise rapidly between populations which are fixed for sex chromosomal variants.

Lineages with multiple-X chromosome systems may be especially strongly affected by these evolutionary regimes because a great proportion of their total genome is captured in the sex chromosomes. The large size of these X chromosomes and their limited heterochromatin content in karyotypic analyses suggest that they contain a large proportion of genes. Multiple-X chromosomes occur widely in vertebrates and invertebrates, for example in monotremes (*Platypus*) [14], howler monkeys (*Aloutta*) [15], fishes [16], gastropod molluscs [17,18], spiders [19], Lepidoptera [20] and several groups of Coleoptera [21-24]. Among these, the widely conserved XXY system in araneomorph spiders [19] and the ancestrally XXXY system in Cicindelidae (tiger beetles) are the most ancient multiple-X system known [25]. Novel X chromosomes of this kind originate readily as a consequence of karyotypic rearrangements involving a sex chromosome and an autosomal pair, producing chiasmatic sex chromosomes. For example, a fusion of the X chromosome with an autosome will produce an expanded X chromosome while the remaining sister chromosome will become an additional sex chromosome. In XY systems, this will produce XXY, whereas in the case of an X0 karyotype, this fusion will produce an enlarged X chromosome and the homolog will act as Y chromosome (producing a neo-XY system). An alternative pathway is that rare chromosomal rearrangements with the autosomes increase the size of the X chromosomes, followed by their dissociation to produce multiple X chromosomes [12]. In all cases, the consequence is that genes are shifted from an autosomal to a sex chromosomal position, and hence alter the evolutionary regime experienced by these genes.

However, the effects of autosomal-heterosomal rearrangements on the evolution of gene regions and recombination-suppressed speciation depend on the evolutionary dynamics and genomic extent of such rearrangements. An unknown factor is what is the frequency of autosomal-heterosomal changes, and if particularly gene regions would be affected repeatedly in the evolution of a lineage. While several studies have assessed these phenomena in populations and closely related species to investigate the microevolutionary consequences of rearrangements [1], only a few studies have addressed these questions on a macroevolutionary (between-species) scale [26]. Here, we conducted a survey of X chromosome numbers and rDNA localization on a representative sample of North American tiger beetles in the genus *Cicindela*. This group exhibits a conspicuous, non-chiasmatic multiple-X chromosome system consisting of 2 to 4 X chromosomes, plus a single Y, and between 9 to 11 pairs of autosomes. The multiple-X system in Cicindelidae is apparently ancient and is present widely in this families except for a set of basal lineages exhibiting an XY system [25]. Despite the presumed antiquity of the system, secondary reversion to simple XY or X0 genetic systems have occurred, but are rare [25,27]. In addition to the karyotypic variation in sex chromosome numbers, species of *Cicindela *differ in the number and localization of rDNA loci which include either two or four pairs of clusters and which are localised on either the autosomes or heterosomes [23,28,29].

The *Cicindela *of the North American continent constitute at least four distinct radiations, each of which with an estimated origin of several million years ago. Based on a phylogenetic tree available at the species level for each of these four lineages [30], the character transitions in sex chromosome number and switches in the position of rDNA between sex chromosomes and autosomes can be analyzed. Information on the tempo and mode of rearrangements in this genetic system that retains a large proportion of the physically observed karyotype in multiple X chromosomes, will provide the backdrop for assessing the effect of autosomal-heterosomal turnover on the evolution of coding regions and the origination of species.

## Results

### X chromosome numbers and rDNA localization

**Figure 1:**
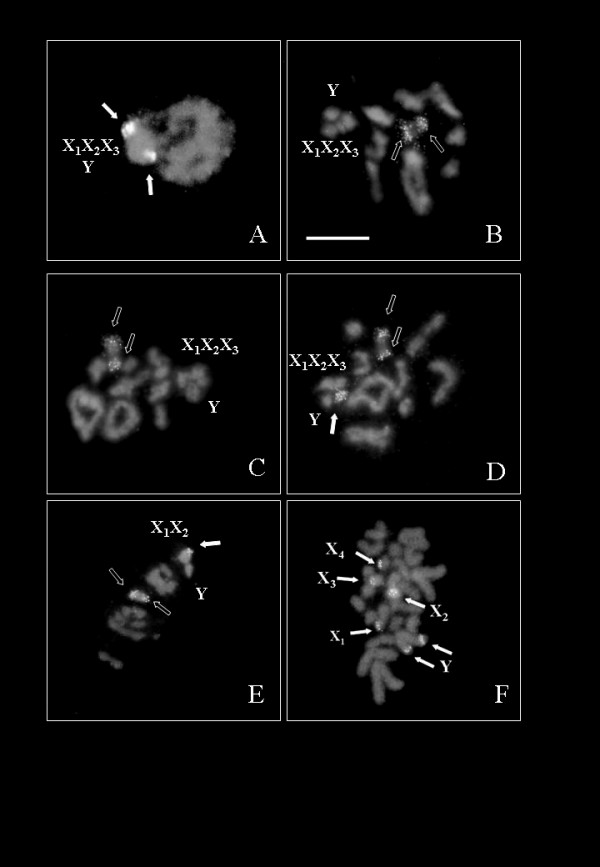
**Fluorescence *in situ *hybridization of tiger beetle chromosomes with a ribosomal probe**. **A**. *Cicindela punctulata*, n = 9 + XXXY, early prophase I nucleus showing two rDNA loci, one located on the Y and another located on one of the four X chromosomes. **B**. *C. scutellaris*, diakinesis with n = 9 + XXXY showing two rDNA loci located on an autosomal pair. **C**. *C. repanda*, diakinesis with n = 9 + XXXY showing two rDNA loci located on an autosomal pair **D**. *C. tranquebarica*, diakinesis with n = 9 + XXXY showing three rDNA loci, two located on an autosomal pair and one located on one of the 3 X chromosomes. **E**. *C. ocellata*, metaphase I with n = 9 + XXY showing two rDNA loci located on an autosomal pair and one on the X chromosome. **F**. *C. marginata*, spermatogonial metaphase with 2n = 23 chromosomes showing six rDNA loci, one located on each of the four X chromosomes and two loci located on the Y chromosome. The white arrows indicate the heterosomal loci, the empty arrows indicate the autosomal loci. The arrowheads point to the sex chromosomes which are condensed in Fig. 1D forming the sex vesicle, and are recognizable individually forming the rosette-like structure in Figs. 1A, B, C, and E.

Karyotype analysis and chromosomal localization of rDNA clusters was carried out in 26 North American species, sampled to cover the main phylogenetic groups of the continent [30]. Examples of karyotype and FISH visualization are shown in Fig. 1, and the results for all species are summarized in Table 1. Autosome numbers were constant for all species tested (n = 9). Heterosome numbers were variable, but most species exhibited an X_1_X_2_X_3_Y karyotype, which is also the most common type in *Cicindela *from the Palearctic, Australia and India, and presumably represents the ancestral state for the entire lineage that also includes other genera [25]. We also found the X_1_X_2_X_3_Y genetic system in several species which in the older literature were reported to exhibit a single X chromosome (*C. pimeriana *[31]; *C. punctulata, C. purpurea*, and *C. repanda *[32]; or X_1_X_2_Y and n = 9 for *Cicindela repanda, C. tranquebarica, C. scutellaris and C. sexguttata *[32], but some of the X chromosomes may have been overlooked in these studies. Our analysis found four species each showing X_1_X_2_X_3_X_4_Y (*C. lemniscata, C. marutha, C. nigrocoerulea *and *C. marginata*) or X_1_X_2_Y (*C. sedecimpunctata*, *C. ocellata*, *C. rugatilis*, *C. nebuligera*) (Table 1). For the autosomes, pairs of homologous chromosomes in meiosis could be paired easily by their size. The autosomal karyotype in all species had many features in common, even where heterosome numbers differed. For example, the largest pair was metacentric and clearly distinguishable from the rest. The second and third pairs were also recognizable as metacentric whereas the fourth was submetacentric. From the third pair to the ninth the size decreased gradually (not shown).

**Table 1:** Karyotypic data of North American species of the genus *Cicindela*

Species	Meioformula	rDNA localization
***Cicindela***		
*duodecimguttata*	9+ X_1_X_2_X_3_Y	Autosomes
*formosa generosa*	9+ X_1_X_2_X_3_Y	Autosomes
oregona*	9+ X_1_X_2_X_3_Y	--
*pimeriana*	9+ X_1_X_2_X_3_Y	Autosomes
*repanda*	9+ X_1_X_2_X_3_Y	Autosomes
*sexguttata*	9+ X_1_X_2_X_3_Y	Autosomes
*splendida*	9+ X_1_X_2_X_3_Y	Autosomes
***Cicindelidia***		
*aterrima*	9+ X_1_X_2_X_3_Y	Heterosomes (XY)
*flohri*	9+ X_1_X_2_X_3_Y	Autosomes
*nebuligera*	9+ X_1_X_2_Y	Autosomes
*nigrocoerulea*	9+X_1_X_2_X_3_X_4_Y	Heterosomes (XY)
*obsoleta*	9+ X_1_X_2_X_3_Y	Autosomes
*ocellata*	9+ X_1_X_2_Y	Autosomes + Heterosomes (X)
*punctulata*	9+X_1_X_2_X_3_Y	Heterosomes (XY)
*roseiventris mexicana*	9+ X_1_X_2_X_3_Y	Autosomes + Heterosomes (X)
*rufiventris*	9+ X_1_X_2_X_3_Y	Autosomes
*rugatilis*	9+ X_1_X_2_Y	Autosomes + Heterosomes (X)
*sedecimpunctata*	9+ X_1_X_2_Y	Autosomes
***Cylindera***		
*hemichrysea*	9+ X_1_X_2_X_3_Y	Autosomes
*lemniscata*	9+X_1_X_2_X_3_X_4_Y	Heterosomes (XY)
***Ellipsoptera***		
*marginata*	9+X_1_X_2_X_3_X_4_Y	Heterosomes (XXXXY)
*marutha*	9+X_1_X_2_X_3_X_4_Y	Heterosomes (XY)
***Habroscelimorpha***		
*dorsalis*	9+ X_1_X_2_X_3_Y	Autosomes + Heterosomes (X)
*fulgoris*	9+ X_1_X_2_X_3_Y	Autosomes + Heterosomes (X)
*severa*	9+ X_1_X_2_X_3_Y	Autosomes + Heterosomes (X)
***Pachydela***		
*scutellaris*	9+ X_1_X_2_X_3_Y	Autosomes
***Tribonia***		
*tranquebarica*	9+ X_1_X_2_X_3_Y	Autosomes + Heterosomes (X)

*Reference 38

We found substantial variation in the number and localization of rDNA clusters (Table 1; Fig. 1). Three types of rDNA constellations were evident, including: localization on the autosomes (one autosomal pair) in about half of the species; localization on the heterosomes (one of the X chromosomes and the Y) found in six species; and localization on both (one autosomal pair plus heterosomal copies located on one of the X chromosome) in the remaining five species. An additional type was found in *C. marginata *where mitotic metaphases showed six rDNA stains on five chromosomes (Figure 1F), with a single chromosome carrying rDNA clusters on both arms. We identified this chromosome as the Y, due to its morphology and orientation on first meiotic plates. In this image the fluorescent signal was located exclusively on the sex vesicle. Whereas all rDNA clusters present in the genome can be detected with FISH, silver staining on spermatogonial cells only shows those genes that are actively transcribed during spermatogenesis. Results from FISH and silver staining were similar generally, indicating that most rDNA clusters were active, but in species with rDNA clusters found on both heterosomes and autosomes, expression was mostly limited to the heterosomal locus, indicating the functional importance of the sex-chromosome linked copies.

### Character reconstruction and covariation

**Figure 2:**
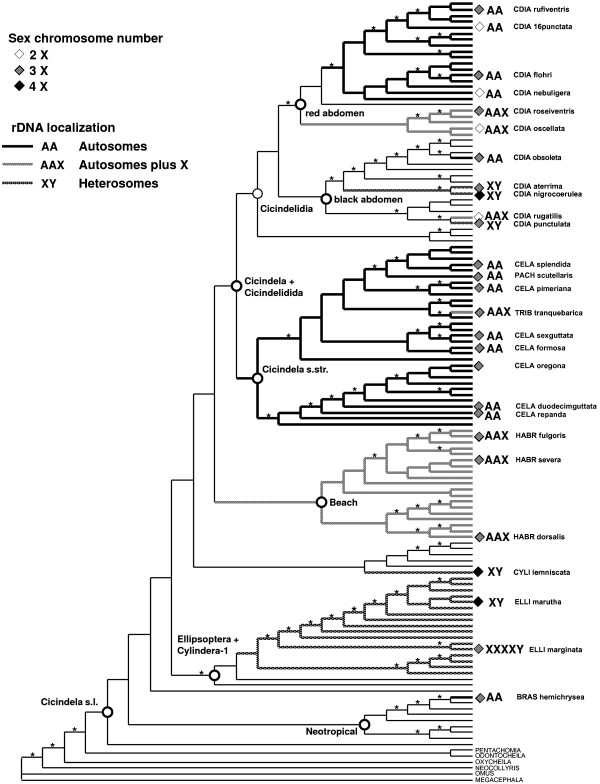
**Phylogenetic distribution of character states in sex chromosome number and rDNA localization. **Character optimization using parsimony was conducted separately for the number of sex chromosomes (2, 3, or 4 X chromosomes) and rDNA localization (localization on autosomes only, on the autosomes and X chromosomes, and on the heterosomes only). The tree is from Vogler et al. (2005) representing approx. 75% of the Nearctic species of *Cicindela* (s.l.) and species names are preceded by an abbreviated subgenus label (CDIA, *Cicindelidia*; CELA, *Cicindela*; TRIB, *Tribonia*; PACH, *Pachydela*; HABR, *Habroscelimorpha*; CYLI, *Cylindera*; ELLI, *Ellipsoptera*; BRAS, *Brasiella*). Several major groupings established in the previous phylogenetic analysis are indicated on the tree. Asterisks above the branches indicate nodes with good support (Bremer Support ≥3 and Bootstrap values ≥60%). Karyotype information is presented by symbols at the tips for all taxa represented in the current study. Different shading of branches indicates the character optimization for rDNA localization. Due to the incomplete taxon sampling it was not possible to assign the character changes to a precise branch deep in the tree, indicated by thin lines.

Parsimony reconstruction (accelerated transformation) on the mtDNA tree inferred the X_1_X_2_X_3_Y system as the ancestral state, with several independent derivations of the X_1_X_2_X_3_X_4_Y and X_1_X_2_Y state (Fig. 2). Whereas the species exhibiting four X chromosomes were found in distant parts of the tree, all species with two X chromosomes were limited to the subgenus *Cicindelidia*. The results were consistent with a repeated transition from X_1_X_2_X_3_Y to either of the two derived states, but not in the reverse direction. The transitions to the X_1_X_2_Y and X_1_X_2_X_3_X_4_Y states were entirely limited to the terminal branches of the tree, and there was no direct transition between these two character states. The localization of rDNA was similarly variable phylogenetically, however the species with exclusively autosomal rDNA (labelled AA in Fig. 2) were largely limited to two clades, the subgenus *Cicindela *(*sensu stricto*) and the 'red abdomen' group of *Cicindelidia*. All other groups exhibited rDNA on the heterosomes, but without phylogenetic conservation of the two main states (autosomal pair plus one X, labelled AAX in Figure 2; or autosomal pair plus one X and Y, labelled XY).

Variation in X chromosome numbers and rDNA localization was largely independent. For example, ten sister species pairs can be assessed for covariation between rDNA positional changes and differences in X chromosome numbers on the tree of Figure 2. Between the sister species a character change in X chromosome number was observed six times, a change in rDNA localization was observed three times, and three species pairs were invariable. Among those sister pairs that were variable for one parameter, a transition in the other parameter is inferred only in two cases, and leading to different states. This demonstrated a broad lack of co-variation indicating that the chromosomal rearrangements producing variation in both karyotypic features were not connected.

## Discussion

Phylogenetic character reconstruction at the species level is useful to test for trait changes associated with speciation events [33]. The incompatibility of rearranged chromosomes could be an important factor leading to reproductive isolation and speciation, in particular where such changes are linked to the sex chromosomes. The genus *Cicindela *is very species rich, which is usually ascribed to ecological shifts in habitat associations [34,35] and a tendency for the isolation of local populations [36,37]. However, karyotypic features have not previously been considered to affect species diversification in this group. Karyotype analyses in *Cicindela *are technically challenging, due to the small size of chromosomes and the absence of the polytene chromosomes common in Diptera, plus the difficulties of obtaining meiotic cells (where multiple sex chromosomes can be accurately identified) except during a short season of the year. Yet, our sampling was sufficiently dense for a comparative analysis. We found that the multiple sex chromosomes provide an evolutionarily dynamic system affected by repeated gains and losses of X chromosomes, and repeated shifts in the localization of the rDNA loci between autosomes and heterosomes, with potential consequences for speciation.

Mechanistically, these shifts in X chromosome numbers do not appear to involve reciprocal rearrangements in the autosomes, as the latter remain invariable, arguing against a mechanism for the origination and loss of X chromosomes through fusions with autosomes. The independence of autosome and heterosome numbers has previously been established in lineages of *Cicindela *in the Palearctic, India and Australia, although in those cases changes affected mainly the autosome numbers while heterosomes were invariable for the modal X_1_X_2_X_3_Y system [38,39]. The absence of autosomal-heterosomal fusions and fissions was also supported by the fact that we did not observe any physical associations of a chiasmatic type between autosomes and heterosomes during meiosis which would be required for such rearrangements. Instead, in all cases studied here the multiple X chromosomes and the Y were connected at the telomeric ends during pairing in male meiosis and were visible as a conspicuous multivalent where (Fig. 1), as had already been described for the Palearctic species *C. hybrida *[40].

The lack of chiasmatic associations between heterosomes and autosomes also argues against a role of fusions and fissions to be responsible for the positional changes of the rDNA clusters. The number and position of rDNA loci can readily be altered by Robertsonian changes (fissions or fusions) via terminal nucleolus organizer regions (NOR) usually containing the rDNA clusters. Two chromosomes each carrying a terminal NOR may either fuse to produce an interstitial NOR, or a single chromosome with an interstitial NOR may undergo fissions resulting in two chromosomes with terminal NORs. However, except perhaps for the case of *C. marginata *with six rDNA loci, we did not see the expected changes in number and position of rDNA loci, as changes in chromosome number and rDNA loci were independent. Therefore neither fissions nor fusions at NORs are supported, unless one invokes additional rearrangements such as pericentric inversion to accommodate the predominantly mediocentric chromosomes in *Cicindela*. Furthermore, considering the achiasmatic nature of heterosomes, Robertsonian rearrangements cannot explain the changes that occur simultaneously in autosomes and heterosomes.

An alternative scenario are non-reciprocal translocations affecting the rDNA clusters which could lead to changes in position (translocation) or numbers of rDNA sites (transposition retaining a copy at the origin). These changes can be facilitated by the presence of transposable elements, as e.g. in the Type I and II ribosomal gene insertions in *Drosophila melanogaster*, *Bombyx mori *[41], *Apis mellifera *and other Hymenoptera [42]. Translocations between chromosomes bearing rDNA loci and those lacking them have been invoked to explain the rearrangements observed in *Paeonia *[43] and *Allium *[44], and also may occur in the ground beetle genus *Zabrus *which presents the highest variability in the number of rDNA sites (2–12) found so far in insects [45]. However, these rearrangements occur among autosomes only, not affecting the sex chromosomes. The peculiar situation in *C. marginata*, where translocation of rDNA copies between chromosomes and even within a chromosome may be responsible for the unusual number of rDNA loci, might be a good model for elucidating the cytogenetic basis for the changes in number of X chromosomes and gene content.

Whatever the mechanisms that produce the change of number and position of rDNA clusters, they seem to be affected by various constraints to the rearrangements of karyotypes. Careful inspection of the chromosome preparations suggested that the rDNA loci were not always in the same pair of autosomes, and may be localized on different homeologous pairs, even in closely related species. Therefore, numerous additional chromosomal rearrangements between autosomes and heterosomes may be present which are not detected here when scoring chromosome numbers and rDNA autosomal-heterosomal localization only. Yet, the overall gestalt of the karyotype was maintained despite these apparent changes in gene content. Nine pairs of autosomes were found in all species, showing very similar sizes and phenotypes. This degree of conservation appears to be specific to the multiple-X karyotype, as such constraints on chromosome morphology are not seen in the ancestral simple-X system of distantly related cicindelids which differ greatly in chromosome number and overall morphology [25]. Similarly, there are apparent constraints to the number and gene content of heterosomes. For example, the X_1_X_2_Y and X_1_X_2 _X_3 _X_4_Y karyotypes represented derived states which were confined to a single species (or perhaps small clades, had denser taxon sampling been available), indicating that deviations from the modal X_1_X_2_X_3_Y system are evolutionarily short-lived and unstable. Finally, the chromosomal positions of rDNA clusters also appeared to be constrained in the multiple-X system. The autosomal number of clusters was two (one pair of allelic copies), but never exceeded this number, and when more than two rDNA copies were found in *Cicindela *they always appeared on the heterosomes, most commonly on the X only, and in some species a further rDNA copy on the Y chromosome. This is in contrast to the single-X chromosome systems in the basal groups of Cicindelidae which exhibit between four and eight (two to four pairs) autosomal rDNA clusters [25]. The nature of these constraints on the karyotype remains unknown but they may be linked to the evolutionary stability of this multiple-X system [25].

Despite the morphological conservation of the multiple-X system, the frequent movements of genes between autosomal to heterosomal positions expose the affected loci to greatly altered dynamics of gene evolution and recombination. As there is no cross-over in the male heterosomes in cicindelids, rates of homologous recombination in the sex chromosomes are reduced by half for the X chromosomes (recombination only in females) and to virtually zero for the Y (resulting in their inevitable degradation; [13]). The hemizygous nature of the X will greatly increase selection on recessive mutations, altering the rate and kind of mutational changes. This would cause the rearranged genes to diverge quickly, even if rearranged gene regions are duplicated. In the case of the rDNA clusters, this could reduce the rate of homogenization of copies in different parts of the genome. For example, an analysis of sequence variation in the ITS1 region of the rDNA cluster in *C*. *dorsalis*, a species shown here to exhibit rDNA copies on a pair of autosomes plus a single copy on the X, exhibited greatly divergent ITS types, possibly corresponding to heterosomal and autosomal copies [46]. This supports the idea that the translocation to the sex chromosomes results in changes of evolutionary dynamics.

These kinds of chromosomal rearrangements might also have an effect on speciation. Translocations of genes between autosomes and sex chromosomes will greatly change the possibilities for gene flow, and changes in the number of X chromosomes may alter the production of balanced gametes. Incompatibility of gametes with different numbers of X chromosomes, or indeed changed position of rDNA clusters, could lead to incorrect separation of chromosomes during anaphase I in a hybrid, and produce a number of unbalanced gametes resulting in reproductive disadvantage. In the case of changes in X chromosome numbers this effect could be exacerbated by altering the sex determination control and the gene regulation associated with changes in heterosome number. As pointed out in the recent literature [1,6,7,47], it is not likely that these 'underdominant' variants become established in a population. This has provided a strong argument against the stasipatric model of speciation [5] which suggests that chromosomal rearrangements cause reproductive isolation due to hybrid dysfunction. However, when involving sex chromosomal unidirectional rearrangements as those in *Cicindela*, this model may still be valid. Depending on the precise genotypes participating in a mating, the combination of certain gametes could lead to a significant proportion of inviable (e.g., no rDNA clusters, unbalanced number of X chromosomes) zygotes, selecting against heterozygotes and maintenance of polymorphisms in a population. These effects may be exacerbated by the reduction of gene flow from suppressed recombination and extending the effect of linked isolation genes, a mechanism proposed as the main driver for the fixation of novel karyotypes under more recent models [8,48]. As gene flow is more restricted between sex chromosomes than autosomes, sex linked genes are particularly efficient to produce such postzygotic barriers [49], and hence rearrangements involving sex chromosomal portions of the genome may be a particularly effective isolating mechanism.

Therefore, the high level of apparent chromosome rearrangements and the deposition of a substantial portion of the genome in the low-recombining sex may promote speciation in *Cicindela*. With some 1,000 species world-wide, this is one of the largest genera of insects. In particular, the observation of evolutionarily short-lived X_1_X_2_Y and X_1_X_2_X_3_X_4_Y lineages suggests that these chromosomal changes could initiate reproductive isolation. If these rearrangements are frequent relative to other (ecological or geographical) processes influencing speciation rates, the cytogenetic parameters could drive speciation and possibly be responsible for the great species richness in *Cicindela*. In addition, the population structure of *Cicindela *is also favoring the fixation of chromosomal mutations locally, as most species are early succession specialists frequently occurring in isolated habitat patches where a dynamic system of colonization and extinction may enhance the separation of local genetic entities. In support of this possibility, we found two Iberian species, *C. flexuosa *and *C. littoralis*, where in each case a single population was fixed for an rDNA localization different from all other populations [29], indicating that local genetic races arise frequently.

## Conclusion

The evolutionary significance of elaborate multiple-X systems compared with simpler sex chromosomes is still poorly understood. Phylogenetic approaches can greatly increase the power of comparative cytogenetic analyses, and have revealed the great fluidity of the cicindelid multiple-X chromosomes, while also establishing evolutionarily conserved features. Even more variable than the X-chromosome numbers are translocations of the rDNA clusters. This provides the background for future investigations to understand the evolutionary forces operating on the sex chromosomes. Whereas the current study uses a macroevolutionary approach, establishing the framework of character variation over greater evolutionary distances, this can be combined with the specific effects of rearrangements on the population level.

Sex chromosomes are of specific interest to speciation, not least because they have been shown to accumulate genes determining species specific traits such as host plant use and pheromone response in butterflies [50]. The observed cytogenetic phenomena should also be studied because of their functional consequences, with regard to the control of sex determination, chromosome size and morphology, and the mechanisms of gene repositioning. Further investigation will require a targeted approach to the comparative genomics of tiger beetles and the construction of chromosomal homology maps, using reciprocal chromosome painting with sex chromosome specific probes obtained by microdissection. Molecular cytogenetics studies, assisted by comparative genomics and phylogenetics, may help to investigate the evolutionary dynamics of gene content of chromosomes and may reveal karyotypic changes that remain unnoticed in conventional cytogenetics analysis. These studies are the basis for tests of how variation in sex chromosomes can drive population differentiation and speciation rates.

## Methods

### Samples for study and chromosome preparations

Taxon sampling for this study was limited to *Cicindela *from North America. There are some 147 described species recorded for the North American continent, grouped in 11 subgenera [51]. A recent phylogenetic analysis based on 1897 base pairs of mtDNA and taxon sampling which is 75% complete at the species level, revealed that most species can be ascribed to four monophyletic groups representing radiations endemic to North and Central America [30]. A representative sample of all major clades in the mtDNA tree was selected for chromosome analysis, with good representation of all four endemic radiations. Character variation in X chromosome number and rDNA localization was assessed on this tree, using parsimony optimization as implemented in MacClade [52]. Adult beetles were obtained in the field, and chromosome preparations were obtained from male gonads. Mitotic and meiotic chromosomes can be obtained at different stages of development in the tubular testes, and can be observed as described previously [28].

### Silver staining and *in situ *hybridization

Active NORs (i.e., regions of active rRNA transcription) were detected with silver according to the [53] technique, with slight modifications [29]. *In situ *hybridization was performed as previously reported in Proença and Galián (2003) using a 555 bp rDNA probe obtained after PCR amplification of the conserved 18S rRNA gene region from *C. campestris *total genomic DNA using the universal primers NSI and NSII [54], labelled with biotin-11dUTP by a second PCR reaction.

## Authors' contributions

JG carried out the design of the study, the collecting of most samples, the molecular cytogenetic analysis, and drafted the manuscript. SJRP participated in the cytogenetic studies. APV conducted the character analysis, and participated in the design of the study and in the writing of the manuscript. All authors read and approved the final manuscript.
